# Investigating the Behavior of Waste Alumina Powder and Nylon Fibers for Eco-Friendly Production of Self-Compacting Concrete

**DOI:** 10.3390/ma15134515

**Published:** 2022-06-27

**Authors:** Safeer Abbas, Malik Asad Ali Ishaq, Syed Minhaj Saleem Kazmi, Muhammad Junaid Munir, Shahid Ali

**Affiliations:** 1 Civil Engineering Department, University of Engineering and Technology, Lahore 54890, Pakistan; safeer.abbas@uet.edu.pk; 2 Civil Engineering Department, National University of Computer and Emerging Sciences (FAST-NU), Lahore 54770, Pakistan; malikasadawan1@gmail.com (M.A.A.I.); shahid.ali@nu.edu.pk (S.A.); 3 School of Engineering, RMIT University, Melbourne, VIC 3001, Australia

**Keywords:** self-compacting concrete, waste alumina powder, nylon fiber, flow

## Abstract

Self-compacting concrete (SCC) incorporating secondary raw materials has been extensively used around the globe due to its improved fresh, mechanical and durability properties. This study was planned to evaluate the suitability of locally available waste alumina powder (AP) and nylon textile fibers (NF) as a partial replacement for fine and coarse aggregates with the ultimate goal to locally produce SCC with desired properties. The used AP was acquired from a local market and NF was collected from a local textile factory. Various dosages of AP (10%, 20%, 30%, 40% and 50% by volume of fine aggregates) and NF (1% and 2% by volume of coarse aggregates) were studied. Tests including slump flow, V-funnel and J-ring tests were performed for examining the fresh properties of developed SCC. Results showed that the addition of AP has an insignificant effect on the superplasticizer dosage for maintaining a constant flow of 70 cm. However, a higher dosage of superplasticizer was required for a mixture with increasing dosages of NF to sustain a constant flow. Similarly, slump flow time (for a spread of 50 cm) and V-funnel time increased for mixtures with higher dosages of AP and NF. Tested SCC mixtures incorporating 40% and 50% of AP with 1% and 2% of NF showed an extreme blocking assessment due to their increased interparticle friction, the higher water absorption capacity of used AP and NF leading to increased flow resistance and hence, showed lower passing ability. The compressive strength was 16% higher for specimens incorporating 40% of AP due to the filling effect of AP which fills the micro-pores, leading to a more dense and compact internal micro-structure, confirmed through scanning electron microscopy analysis. An ultrasonic pulse velocity test conducted on hardened specimens verified the findings of the compressive strength results. Moreover, it was observed that NF has an insignificant effect on the compressive strength; however, flexural strength was increased due to the incorporation of NF, especially at higher dosages of AP.

## 1. Introduction

Concrete is an important building material and has been commonly used in the construction industry all around the world [1,2]. With the passage of time, significant changes in the structure of concrete have been observed. These advance changes in structure have led to the gradual replacement of conventional concrete with recent and more up-to-date concrete such as polymer-modified concrete, special concrete incorporating various waste materials and self-compacting concrete (SCC) among others. SCC is a self-flowable non-segregating concrete that does not require any mechanical vibration for its consolidation and can easily fill the molds even in the case of dense reinforcement [3,4]. Okamura and Ouchi [5] first developed the mixture for SCC after facing prominent durability problems with conventional concrete. The main conclusions of their research work stated that concrete did not require any sort of vibration in order to achieve full compaction and flowed under its own weight [5,6]. The fundamental requirements of SCC as regards its state are described thus: it should be self-compactable in its fresh form; it should be resistant to bleeding and segregation; at early stages it should be resistant to shrinkage and it should give high long-term and durability performance [5,6,7,8].

Self-compacting concrete turned out to be a milestone in the age of progressive technology and is extensively used in the construction field due to its various benefits. In the modern age, many working policies and platforms in construction companies have limitations; however, SCC is an excellent substitute for traditional high slump concrete. SCC outperforms enormous settlements in heavily reinforced sectors where its major use is for transportation structures, pre-stressed concrete members tunnel linings, rafts and pile foundations, high-rise buildings, the repair of structures such as aircraft runways and bridge piers [9,10,11,12]. SCC has the self-ability of incorporating secondary raw materials which makes it an acceptable material worldwide and increases its significance [13,14,15].

Concrete, being the foremost construction material used extensively around the globe, is the principal source involved in the emission of carbon dioxide (CO_2_). Due to the extensive use of concrete, several environmental aspects are at risk as it involves the release of CO_2_. Several building materials and concretecontaining clinker or stony residues from burnt coal are the main components involved in carbon dioxide as 1 ton of cement is equivalent to the release of 0.9 to 1.3 tons of CO_2_ in the surroundings [16]. In order to overcome CO_2_ production due to increased construction activities, it has been proposed to partially replace the cement in concrete with finely ground additives such as slag, silica fumes, fly ash or other pozzolanic materials. Various studies have been conducted in the past to evaluate the effectiveness of pozzolanic materials on the properties of SCC. For example, Rizwan and Bier [17] studied the effective impact of size, surface morphology, porosity and shape of secondary raw material on the superplasticizer demand and water requirement of a developed SCC system. It was reported that material with high porosity such as rice husk ash showed higher water adsorption and materials with an increased surface area resulted in lesser demand for plasticizer [17]. Hameed et al. [18] studied the effect of waste marble powder (WMP) on the properties of SCC and concluded that 15% of WMP is the optimum dosage for controlling the fresh and hardened properties of tested SCC mixtures. Neelam and Siddique [19] studied the properties of SCC for a mixture incorporating fly ash. Satish et al. [20] concluded that the spread flow increased for a mixture incorporating a higher dosage of fly ash. Similarly, Cabrera et al. [21] studied the use of biomass bottom ash for determining the fresh and hardened properties of self-compacting concrete. De Matos et al. [22] used an air-cooled blast furnace slag and limestone filler for the production of SCC with desired properties. All the tested SCC mixtures showed a slump flow of 650 mm [22]. Moreover, the utilization of nanomaterials, quarry wastes or other filler materials enhanced the fresh, mechanical and durability properties of concrete [23,24,25,26]. Therefore, it can be argued that the appropriate combinations of secondary raw materials and nanomaterials/filler materials can result in an improved self-compacting concrete system with the desired fresh, mechanical and durability performance.

Various studies have been conducted in the past on the use of waste alumina powder (AP) and nylon fibers (NF) for improving the conventional concrete properties [27,28,29,30]. However, limited studies are available in the open literature on the combined use of locally available AP and NF in exploring the fresh and mechanical properties of SCC. Therefore, this study aimed to examine the various dosages of AP (10%, 20%, 30%, 40% and 50% by volume of fine aggregates) and NF (1% and 2% by volume of coarse aggregates) on fresh and hardened properties of SCC. This study will facilitate various stakeholders and investors in utilizing the waste materials (AP and NF) in construction activities, leading to more durable, eco-friendly and sustainable infrastructures.

## 2. Materials and Mixture Proportions

Ordinary Portland cement (OPC) of Grade 42.5 conforming to ASTM C150 [31] was used during this research program. The average particle size of used cement was 21 µm and the water demand was 25.5%. The specific gravity and surface area of used cement was 3.11 and 1630 cm^2^/g. Table 1 shows the chemical properties of used cement. Locally available sand and crush was used in this research project for fine and coarse aggregates, respectively. Table 2 shows the physical properties of used aggregates. Third-generation high-performance polycarboxylate based superplasticizer in liquid form was used in the present study. Table 3 shows the physical properties of superplasticizer. Ordinary tap water was used in all the tested mixtures.

**Figure 1:**
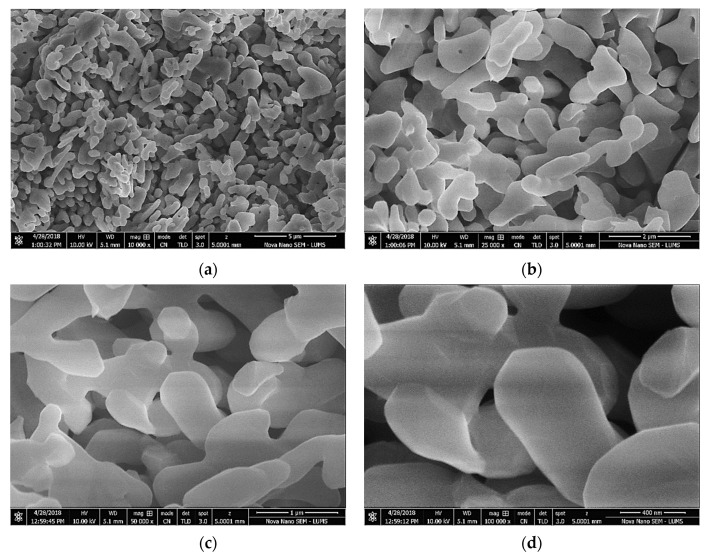
SEM images of waste alumina powder. (**a**) 5 µm (10,000× mag.) (**b**) 2 µm (25,000× mag.) (**c**) 1 µm (50,000× mag.) (**d**) 400 nm (100,000× mag.). (mag. = magnification).

**Figure 2:**
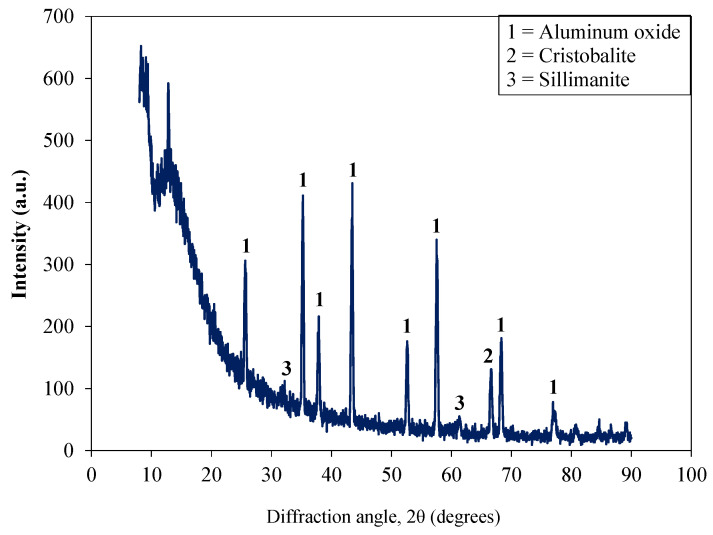
XRD analysis of used waste alumina powder.

Waste alumina powder (AP) was collected from a local factory where a sandblasting operation had been done to give final shape to the products. The procured AP material was then sieved through a BS-410 # 350 sieve. The size of the sieved material was about 8 microns. AP showed a moisture content of 1.28% at 24 h of oven drying at 108 °C. Table 1 also shows the chemical properties of used AP. Scanning electron microscope (SEM) images showed the irregular and rough texture of the used AP (Figure 1). Figure 2 shows the X-ray diffraction (XRD) analysis of used AP. Peaks of aluminum oxide were detected in the XR analysis of AP. Moreover, smaller amounts of cristobalite and sillimanite were also observed. Nylon fiber (NF) is a by-product of polyamides. NF was obtained from a textile factory near Lahore. Wet NF was dried in a laboratory for 20 h at room temperature before its use. The used fiber length was 12 mm.

A high-performance concrete pan mixer was used for mixing purposes. All materials were placed in the pan of a concrete mixer in layers starting with fine aggregates, AP, NF, cement and coarse aggregates. These materials were dry mixed for one minute at 150 rpm. Afterwards, about 75% of the mixing water was added and the mixing was continued for another 5 min. The required SP was mixed in the remaining 25% of water and added to the wet mixture. Mixing was again continued for 5 min at 150 rpm. Afterwards, fast mixing was done for another 5 min at a speed of 300 rpm. Table 4 shows the mixture design tested in this research program.

## 3. Experimental Procedures

Initially, various tests on developed SCC were conducted to evaluate its fresh properties. For instance, slump flow spread was performed in accordance with ASTM C1611 [32] for the judgment of flowability of SCC. The mentioned slump flow diameters were the average value measured at four different locations. After the completion of the mixing process, the concrete mixture was poured into a slump cone. The inner surface of the cone and the test surface of the base plate were wetted using the moist sponge and the cone was placed in the center of the base plate. After filling the cone with concrete mixture, the cone was lifted perpendicularly to the base plate in a single movement and the concrete mixture was allowed to flow freely without any obstruction. The time was noted from the moment the cone lost contact with the base plate. The stopwatch reading was recorded for T_50cm_ time and total spread time. Furthermore, the concrete spread was visually checked carefully for any segregation. If the slump flow measured was lower than the desired value, more superplasticizer was added and the test was repeated until the required slump flow was achieved. A V-funnel test was also performed on the tested concrete mixtures in accordance with EFNARC specifications [4]. A V-funnel was vertically placed. The inner side of the funnel was wetted with a water sponge. The opener of the funnel was closed and a bucket was placed underneath the V-funnel. The funnel was filled with the SCC mixture via a closed gate. The stopwatch was turned on at the moment of the gate opening and the flow time was recorded and reported.

The limited deformability of SCC due to the blocking effect of confined reinforcement bars can be judged by a J-ring flow spread. Therefore, a J-ring test was also performed in accordance with ASTM C1621 [33]. The inner face of the cone and base plate were wetted using the moist sponge. The J-ring was placed on the base plate. The cone was filled with an SCC sample from the mixer and the cone was sharply lifted to the perpendicular base plate, in such a way that the SCC was allowed to flow out with freedom and without impediment from the cone; the stopwatch was turned on for time recording. The time was noted down as the SCC sample approached the diameter 50 cm (T_50cm_). The time of the total spread until the flow stopped was also recorded.

Cylinders of size 150×300 mm were also cast for tested mixtures for evaluating the compressive strength as per ASTM C39 [34]. Small prisms of size 100 × 100 × 500 mm were also prepared for measuring the flexural strength in accordance with ASTM C1609 [35]. After casting, specimens were covered with a plastic sheet to avoid loss of moisture. After 24 h, specimens were taken out from their respective molds and cured in normal water until testing time. Specimens were tested at 7, 14, 28 and 56 days. An ultrasonic pulse velocity test was also conducted on hardened specimens at the desired times, following ASTM C597 [36].

## 4. Results and Discussion

### 4.1. Fresh Properties

#### 4.1.1. Superplasticizer Dosage

**Figure 3:**
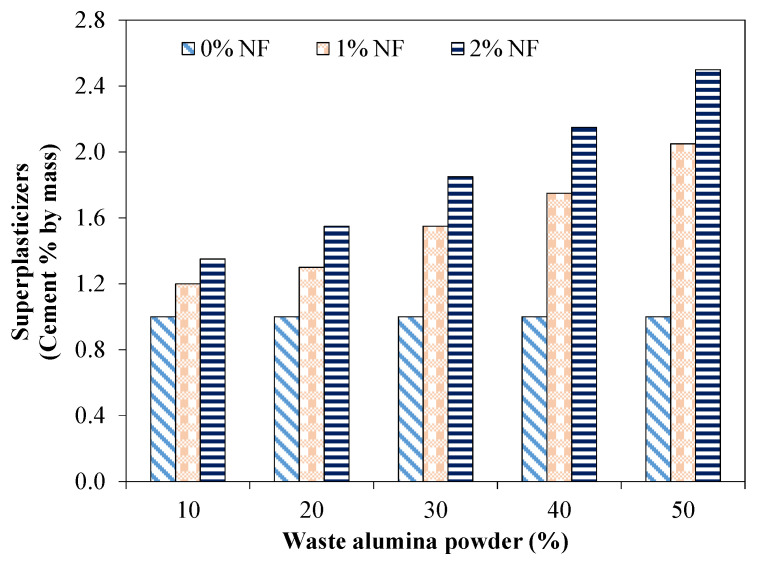
Superplasticizer demand for mixtures incorporating AP and NF.

Figure 3 shows the superplasticizer requirement for achieving the desired slump of 70 cm for a mixture incorporating various proportions of AP and NF. SCC should have characteristics of both high deformability offering lower resistance to flow and high viscosity simultaneously. Using a superplasticizer ensures a high level of deformability, which leads to lower water to cement ratio. The superplasticizer disperses the cement grains, eradicates interparticle friction and ensures reduction in water content while keeping the required levels of flowability and viscosity [37].

From the results, it was observed that the addition of AP had an insignificant effect on the dosage of the superplasticizer for achieving the desired flow for mixture without NF. However, the mixture incorporating nylon fibers showed a higher demand of superplasticizer to attain the desired flow. For instance, a mixture with 1% and 2% of NF required 1.30 and 1.55% by cement mass of superplasticizer, respectively, for a desired flow (Figure 3). In fact, fibers acted as a barrier and restricted the flow leading to a higher demand for superplasticizers to achieve a constant flow. Furthermore, it was evident that the fiber effect was more dominant for the mixture incorporating higher proportions of AP. For example, the mixture incorporating 2% of NF and 50% of AP required 2.5% by cement mass of the superplasticizer compared to that of 1.85% by cement mass of the superplasticizer for the identical mixture with 30% of AP.

#### 4.1.2. Slump Flow Time

**Figure 4:**
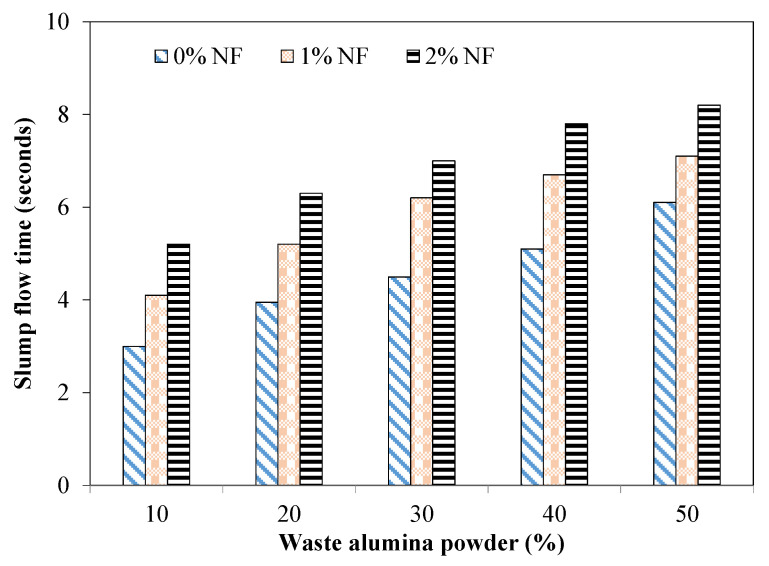
Slump flow time for mixture incorporating AP and NF.

Figure 4 shows the slump flow time for the mixture incorporating various proportions of AP and NF. This flow time corresponds to the time required by the mixture to flow 50 cm. It was observed that the flow time increased due to the addition of AP in the tested SCC mixtures. For instance, the flow time was 3.9 and 5.1 s for the mixture incorporating 20% and 40% of AP without fibers. This increased time due to incorporation of AP was mainly related to the higher water absorption capacity of AP leading to an increase in the viscosity and consequently a decrease in the flowability [38]. Moreover, the increased AP dosage may have led to the lack of interlocking phenomena in between the AP particles and the bond between AP particles and aggregate particles [39]. An increased flow time was observed for mixtures incorporating NF. For instance, the mixture incorporating 30% of AP exhibited a flow time of 4.5 and 6.2 s for the mixture without fiber and 1% fibers, respectively. Moreover, with an increased fiber dosage, an increased flow time was noted owing to the hindering effects provided by the fibers. The acceptance flow time for SCC mixtures is 3 to 7 s [4]. All the tested mixtures without fibers satisfied the limiting value of the flow time for all AP dosages. However, the mixture incorporating 2% of NF with 40% and 50% of AP showed a flow time higher than 7 s.

#### 4.1.3. V-Funnel Test

**Figure 5:**
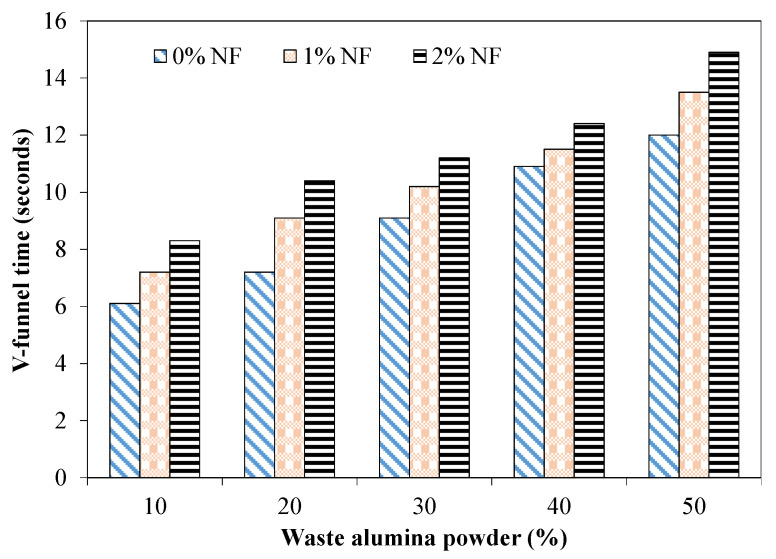
Flow time for mixture incorporating AP and NF.

The V-funnel test measures the concrete resistance against segregation by examining the mixture’s viscosity. Figure 5 shows the flow time through the V-funnel test. This flow time is linearly increased with increased levels of fine aggregate replacement. The flow time was around 9 s for the mixture incorporating 30% of AP which increased to 12 s for mixtures with 50% of AP without fibers. AP particles have the ability to absorb water from the mixtures, leading to more viscous mixtures which hinder the flow and also lead to a reduction in the mixture’s bleeding [39].

It was observed that nylon fibers significantly affect the flow time at all replacement levels of aggregates with AP. For example, the flow time for the mixture with 20% of AP was 10 s with 2% of NF in comparison with 7 s for the identical mixture without fibers. V-funnel flow time can be deemed as satisfactory if it lies between 8 and 10 s [4]. Mixtures incorporating 30%, 40% and 50% of AP without fibers can be considered acceptable as their flow time was within the specified range. Mixtures incorporating 1% and 2% of fibers with 50% of AP showed a flow time higher than 12 s, indicating more viscous mixtures, and did not satisfy the EFNARC requirements [4].

#### 4.1.4. Blocking Assessment

**Figure 6:**
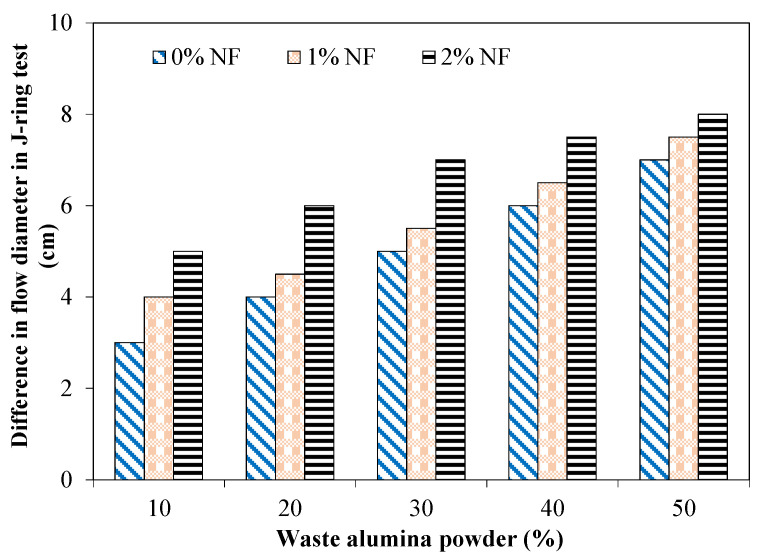
Difference in flow diameter in the J-ring test for mixtures incorporating AP and NF.

In order to evaluate the blocking assessment of various mixtures incorporating AP and NF, a J-ring test was performed and the results are reported in Figure 6. Furthermore, the J-ring test gave an indication of the concrete mixture’s ability to fill the voids and of how easily it flows through the obstructions. The blocking assessment was determined by subtracting the slump flow values from J-ring flow values. It was observed that the flow difference increased for mixtures with higher dosages of AP. For instance, the flow difference was 4 cm and 7 cm for mixtures incorporating 20% and 50% of AP without fiber contents. The addition of nylon fibers further increased the flow difference. For example, the mixture incorporating 2% of fibers showed a 6 cm flow difference with 20% of AP in comparison with a 4 cm flow difference for an identical mixture without fibers. A maximum flow difference of 8 cm was observed for the mixture with 50% of AP and 2% of NF.

No blocking can be assigned for mixtures exhibiting a difference in flow of less than 2.5 cm, and minimum to noticeable blocking if the difference in flow is between 2.5 and 5 cm; extreme blocking can be considered for a flow difference greater than 5 cm [4]. Tested mixtures with 20% and 30% of AP without fibers were in the range of minimum to noticeable blocking. Extreme blocking can be considered for mixtures with 40% and 50% of AP without fibers. Similarly, mixtures with 1% and 2% of fibers and also incorporating 40% and 50% of AP exhibited in the range of extreme blocking. Tested SCC mixtures having 40 to 50% of AP and NF exhibited extreme blocking because of increased interparticle friction, higher water absorbed by AP and fibers leading to higher resistance to flow and consequently lower passing ability.

### 4.2. Hardened Properties

#### 4.2.1. Compressive Strength

**Figure 7:**
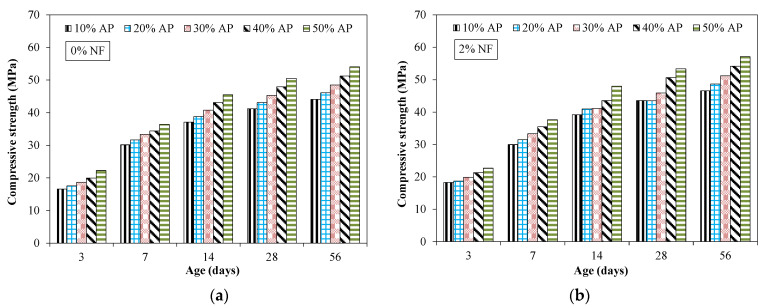
Compressive strength for specimens incorporating AP and fibers. (**a**) 0% fiber (**b**) 2% fibers.

Figure 7 shows the results of compressive strength for tested mixtures. All results reported in Figure 7 showed the average of 3 specimens. Table 5 shows the coefficient of variation (COV) for compressive strength results. For all the tested mixtures incorporating various proportions of NF and AP, the COV was less than 1.5%.

**Figure 8:**
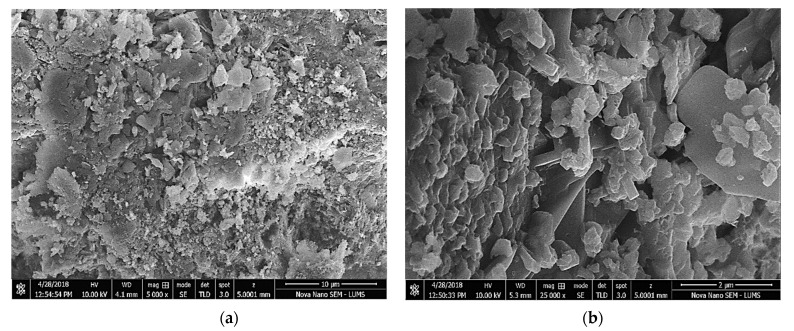
SEM images of specimens incorporating 30% of AP. (**a**) 10 µm (5000× mag.) (**b**) 2 µm (25,000× mag.). (mag. = magnification).

It was observed that the compressive strength increased at later times compared to earlier times for all the tested specimens incorporating AP and NF. This was due to the continuous ongoing hydration process. It was observed that specimens incorporating 10% of AP exhibited a compressive strength of around 16 MPa at 3 days compared to that of 44 MPa at 56 days for an identical mixture. Furthermore, it was observed that the compressive strength increased with increased dosage of AP at all tested times. For instance, at 28 days, the compressive strength was 16% and 23% higher for specimens incorporating 40% and 50% of AP compared to that of the identical specimen with 10% of AP. This increase in compressive strength was mainly attributed to the filling effect of AP which fills the micro-pores leading to a more dense and compact internal micro-structure [38]. Figure 8 shows the SEM images of specimens incorporating 20% of AP, representing the dense and compact micro-structure.

**Figure 9:**
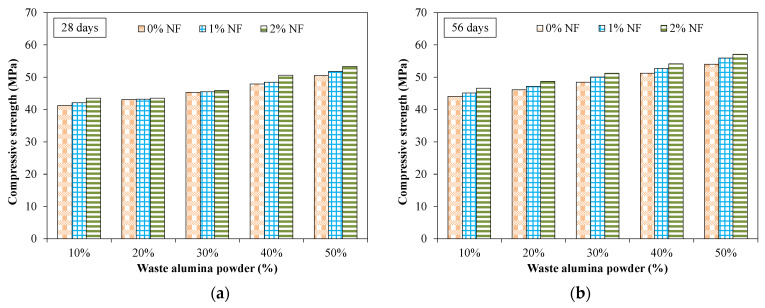
Effect of fibers on compressive strength for specimens incorporating AP. (**a**) 28 days (**b**) 56 days.

Moreover, the alumina powder may act as a pozzolanic material by reacting with calcium hydroxide and also contributed to stronger mechanical interlocking between particles [38]. Figure 9 shows the effect of nylon fibers on the compressive strength for specimens incorporating various dosages of AP. It was observed that there was no significant effect of fibers on the compressive strength at all the tested times. For instance, the specimen incorporating 40% of AP at 28 days showed a compressive strength of around 47 MPa and 50 MPa for specimens without fibers and specimens with 2% of NF, respectively. Maximum compressive strength of around 57 MPa was observed at 56 days for specimens incorporating 50% of AP and 2% of NF.

#### 4.2.2. Flexural Strength

**Figure 10:**
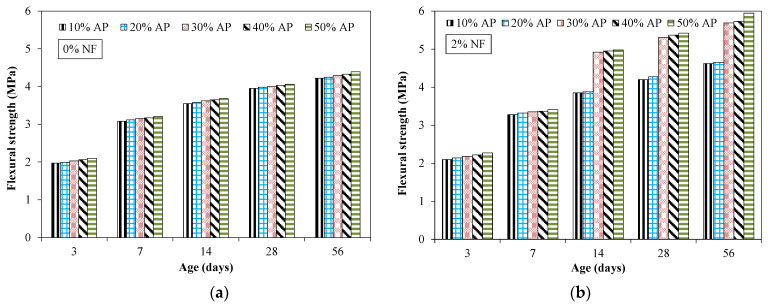
Flexural strength results for specimens incorporating AP and fibers. (**a**) 0% fiber (**b**) 2% fibers.

Figure 10 shows the flexural strength results of specimens incorporating AP and NF. All results reported were the average of three identical specimens. Table 6 shows the COV for the flexural strength results incorporating various proportions of NF and AP.

**Figure 11:**
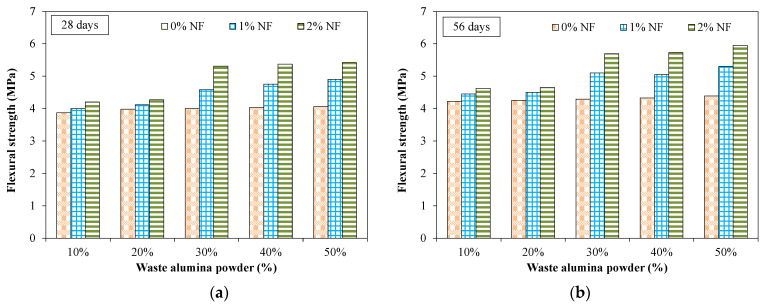
Effect of fibers on flexural strength specimens incorporating AP and fibers. (**a**) 28 days (**b**) 56 days.

An increase in flexural strength was observed with increased curing time. For example, the flexural strength was 3.08, 3.95 and 4.22 MPa at 7, 28 and 56 days, respectively, for a specimen incorporating 10% of AP. It was observed that the dosage of AP had no significant effect on the flexural strength of tested specimens without NF. For example, specimens incorporating 20% and 50% of AP without fibers exhibited flexural strength of 3.98 and 4.06 MPa. It was observed that flexural strength increased due to the incorporation of fibers for all the tested mixtures (Figure 11). For instance, flexural strength was 4.03 MPa at 28 days for a specimen incorporating 40% of AP without fibers and 5.37 MPa for an identical mixture with 2% of NF. This indicates the beneficial effect of NF which restricts the formation of micro-cracks and bridges the developed cracks, leading to a restriction of the further widening of these cracks.

#### 4.2.3. Ultrasonic Pulse Velocity Test

**Figure 12:**
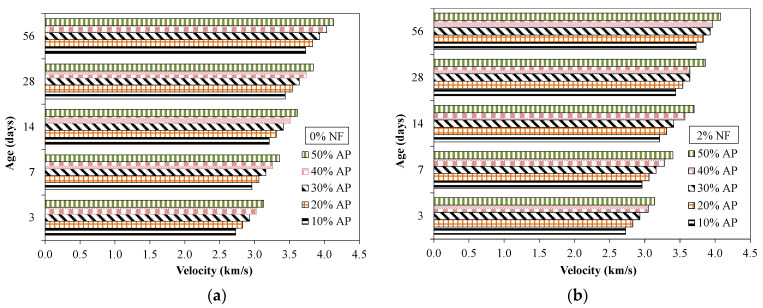
Ultrasonic pulse velocity test results for specimens incorporating AP. (**a**) 0% fibers (**b**) 2% fibers.

Figure 12 shows the results of ultrasonic pulse velocities conducted on specimens incorporating various proportions of AP and NF. Table 7 shows the COV for ultrasonic pulse velocity results. It was observed that the UPV was higher for specimens tested at later times compared to specimens tested at earlier times, in accordance with the compressive strength results. The UPV was 2.96 km/s and 3.73 km/s at 7 and 56 days, respectively, for specimens incorporating 10% of AP. This higher value of UPV at later times was mainly related to the pozzolanic nature of used AP. The UPV increased for specimens incorporating a higher dosage of AP. For example, the UPV was 3.21 km/s and 3.61 km/s at 14 days for specimens incorporating 10% and 50% of AP. This higher value of UPV with a higher dosage of AP was attributed to greater compactness and a dense structure owing to the filling effect of used AP. In fact, AP particles were replaced with sand and being finer material than sand, they filled the micro-pores and voids in between the sand particles, and consequently a more granular skeleton and compact internal microstructure was formed [39,40]. Moreover, no significant effect of NF on the UPV for tested mixtures was observed. The UPV results were consistent with the compressive strength results (reported earlier).

## 5. Conclusions

This study explored the effect of waste alumina powder (AP) and nylon fiber (NF) on self-compacting concrete (SCC). Various dosages of AP (10%, 20%, 30%, 40% and 50%) by volume of fine aggregates were investigated. NF was incorporated in the mixture by 1% and 2% of coarse aggregates. Fresh properties of SCC were investigated by performing a slump test, a V-funnel test and a J-ring test. Mechanical properties of specimens incorporating developed SCC with AP and NF were also examined on hardened specimens.

From the conducted experiments, it was observed that the addition of AP had no significant effect on the superplasticizer dosage for attaining the desired flow of 70 cm. However, the mixture incorporating NF showed a higher demand of the superplasticizer for achieving the desired flow. For instance, mixtures with 1% and 2% of NF required 1.30 and 1.55% by cement mass of superplasticizer, respectively, for the desired flow. It was observed that the slump flow time increased due to the addition of AP in the tested SCC mixtures. For instance, the slump flow time increased from 3.9 s (for the mixture with 20% of AP) to 5.1 s for the mixture incorporating 40% of AP. This increased time due to the incorporation of AP was mainly related to the higher water absorption capacity of AP leading to an increase in the viscosity and a decrease in the flowability. The flow time was increased due to the incorporation of NF. For example, the mixture incorporating 30% of AP exhibited a flow time of 6.2 s for 1% NF compared to that of 4.5 s for mixtures without fibers. All the tested mixtures without fibers satisfied the limiting value of flow time (3 to 7 s). The mixture incorporating 2% of NF with 40% and 50% of AP exhibited a slump flow time higher than 7 s. The J-ring test showed that the tested mixtures with 20% and 30% of AP without fibers were in the range of minimum to noticeable blocking. Mixtures with 1% and 2% of NF and also incorporating 40% and 50% of AP demonstrated a range of extreme blocking. This extreme blocking may be attributed to increased interparticle friction, increased water absorbed by AP and NF leading to higher resistance to flow and consequent lower passing ability.

It was observed that the compressive strength increased with an increased dosage of AP at all tested times. For instance, at 28 days, the compressive strength was 16% and 23% higher for specimens incorporating 40% and 50% of AP compared to that of the identical specimen with 10% of AP. This increase in compressive strength was mainly attributed to the filling effect of AP which fills the micro-pores, leading to a more dense and compact internal micro-structure. SEM analysis confirmed the dense and compact micro-structure of specimens incorporating AP. The UPV test verified the findings of compressive strength results. It was observed that AP had no significant effect on the flexural strength of tested specimens. However, it was evident that the flexural strength increased for specimens incorporating NF. For instance, the flexural strength at 28 days was 5.37 MPa for specimens incorporating 2% of NF compared to that of 4.03 MPa for identical specimens without NF. This indicated the valuable effect of NF in improving the flexural properties of SCC through bridging and arresting the micro-cracks.

## Figures and Tables

**Table 1:** Properties of used cement and AP.

Constituents	Cement (%)	AP (%)
SiO_2_	17.17	2.56
Al_2_O_3_	5.50	91.47
Fe_2_O_3_	3.20	1.08
MgO	1.48	0.05
CaO	64.12	1.16
Na_2_O	1.89	0.54
K_2_O	1.18	0.39

**Table 2:** Physical properties of used fine and coarse aggregates.

Constituents	Fine Aggregate	Coarse Aggregate
Specific gravity	2.88	2.75
Water absorption	1.40%	0.30%
Fineness modulus	2.20	2.60

**Table 3:** Properties of used superplasticizer.

Properties	Description/Values
Color	Light brown
Physical appearance	Liquid
Viscosity	130 cps
pH value	6.7
Relative density	1.2

**Table 4:** Mixture design.

Constituents	Mass/Cement Mass
Cement	1.00
Sand	2.05
Crush	1.78
Water	0.35
AP	##
NF	!!

## 10%, 20%, 30%, 40% and 50% by fine aggregates volume; !! 1% and 2% by volume of coarse aggregate volume.

**Table 5:** Coefficient of variation for compressive strength results.

NF	AP	Compressive Strength (COV, %)				
		3 Days	7 Days	14 Days	28 Days	56 Days
0%	10%	0.90	0.41	0.50	0.82	1.20
	20%	0.42	0.68	0.45	0.32	1.05
	30%	0.50	0.82	0.35	0.95	0.85
	40%	0.93	0.42	0.55	0.80	0.95
	50%	0.97	0.32	0.44	0.75	1.12
1%	10%	0.68	0.21	0.35	0.45	0.80
	20%	0.15	0.35	0.57	0.58	0.30
	30%	0.50	0.48	0.27	0.70	0.74
	40%	0.34	0.29	0.85	0.95	0.85
	50%	0.78	0.35	1.01	1.21	1.05
2%	10%	0.32	0.52	0.35	0.58	1.12
	20%	0.42	0.87	0.89	0.75	0.40
	30%	0.12	0.18	0.15	0.81	0.68
	40%	0.25	0.16	0.24	0.55	0.74
	50%	0.95	0.85	0.78	1.02	0.30

**Table 6:** Coefficient of variation for flexural strength results.

NF	AP	Flexural Strength (COV, %)				
		3 Days	7 Days	14 Days	28 Days	56 Days
0%	10%	0.90	0.30	0.80	0.85	0.75
	20%	0.65	0.42	0.14	0.68	0.52
	30%	0.95	0.51	0.32	0.26	0.38
	40%	0.45	0.25	0.65	0.48	0.65
	50%	0.13	0.12	0.82	0.19	0.16
1%	10%	0.12	0.22	0.35	0.25	0.28
	20%	0.62	0.42	0.68	0.45	0.35
	30%	0.25	0.40	0.38	0.18	0.15
	40%	0.15	0.20	0.18	0.35	0.24
	50%	0.25	0.75	0.63	0.55	0.72
2%	10%	0.70	0.19	0.11	0.20	0.40
	20%	0.10	0.11	0.17	0.72	0.10
	30%	0.18	0.72	0.92	0.13	0.19
	40%	0.21	0.14	0.52	0.18	0.11
	50%	0.13	0.20	0.13	0.21	0.82

**Table 7:** Coefficient of variation for ultrasonic pulse velocity results.

NF	AP	Ultrasonic Pulse Velocity (COV, %)				
		3 Days	7 Days	14 Days	28 Days	56 Days
0%	10%	0.60	0.10	0.18	0.18	0.52
	20%	0.32	0.30	0.15	0.42	0.92
	30%	0.80	0.82	0.18	0.65	0.17
	40%	0.11	0.15	0.92	0.16	0.20
	50%	0.18	0.20	0.10	0.52	0.65
2%	10%	0.70	0.10	0.82	0.10	0.92
	20%	0.10	0.15	0.52	0.19	0.65
	30%	0.16	0.19	0.10	0.14	0.11
	40%	0.18	0.10	0.18	0.21	0.82
	50%	0.65	0.83	0.62	0.14	0.73

## Data Availability

Not applicable.
